# Antiphospholipid antibodies induce endothelial procoagulant activity and release of extracellular vesicles independently of a second hit

**DOI:** 10.3389/fimmu.2025.1702103

**Published:** 2025-12-16

**Authors:** Daniel Álvarez, Hephzibah E. Winter, Udo R. Markert, Ángela P. Cadavid J., Diana M. Morales-Prieto

**Affiliations:** 1 Grupo Reproducción, Departamento Microbiología y Parasitología, Facultad de Medicina, Universidad de Antioquia UdeA, Medellín, Colombia; 2 Placenta Lab, Department of Obstetrics, Jena University Hospital, Jena, Germany; 3 Facultad de Enfermería, Universidad Cooperativa de Colombia (UCC), Medellín, Colombia; 4 Grupo de Investigación en Trombosis, Departamento Medicina Interna, Facultad de Medicina, Universidad de Antioquia UdeA, Medellín, Colombia

**Keywords:** antiphospholipid antibodies, antiphospholipid syndrome, hemostasis, endothelial cells, extracellular vesicles, β2-glycoprotein-I

## Abstract

**Introduction:**

Antiphospholipid antibodies (aPLs) can promote thrombosis *in vivo*, but evidence from both animal models and clinical data suggests that they act as a ‘first hit’ and may require a ‘second hit’, typically an inflammatory stimulus, to induce thrombus formation. This study aimed to investigate whether polyclonal aPLs alone can induce effects in human endothelial cells that are sufficient to trigger *ex vivo* clot formation and to induce the release of endothelial extracellular vesicles (EVs) carrying an altered cargo.

**Methods:**

Human umbilical vein endothelial cells (HUVECs) were stimulated with IgG from patients with vascular and obstetric APS (IgG APS) or IgG purified from the serum of healthy women with proven gestational success (IgG healthy control —HC—). IgG binding to HUVECs, expression of tissue factor, and the procoagulant activity of the endothelial surface were evaluated. EVs were isolated from the supernatants and characterized by nanoparticle tracking analysis, cryo-transmission electron microscopy, flow cytometry, and Western blotting.

**Results:**

Compared to IgG HC, IgG APS showed increased binding to the endothelial surface upon prior and concomitant stimulus with LPS (HC 10.92; APS 81.61 mean fluorescence intensity —MFI—, p < 0.01). This enhanced binding capacity of IgG APS to HUVECs was preserved even in the absence of LPS (HC 12.23; APS 84.26 MFI, p < 0.05). Additionally, IgG APS enhanced the platelet-rich plasma-dependent procoagulant activity of the endothelial surface (HC 0.031; APS 0.098 clot density; p < 0.05), and the release of large EVs (HC 7.3 x 10^8^; APS 1.1 x 10^9^ particles; p < 0.05). These lEVs were frequently opsonized by IgG (lEV_C_ 28.5; lEV_APS_ 40.6%, p < 0.001).

**Conclusion:**

Our findings suggest that IgG APS can trigger second hit-independent procoagulant mechanisms in HUVECs and induce the release of lEVs that subsequently display surface-bound IgG, highlighting a potential role for endothelial EVs in APS pathophysiology.

## 1 Introduction

Antiphospholipid syndrome (APS) is an autoimmune disease mainly characterized by vascular thrombosis and pregnancy-related morbidity, along with the persistent presence of antiphospholipid antibodies (aPLs) (1). The scarce prevalence of thrombosis in patients positive for aPLs with adverse pregnancy outcomes (2), as well as *in vitro* evidence showing the effect of IgG from patients with vascular and obstetric manifestations on monocytes and trophoblast cells (3, 4), suggest the differentiation of APS into two independent entities: vascular APS — driven by a non-inflammatory hypercoagulable state (5) — and obstetric APS — which was earlier explained by the development of placental thrombosis (6) and is currently understood as a result of a localized inflammatory process in the placenta (7, 8).

One of the hypotheses raised to explain this differentiation is referred to as the “two-hit” hypothesis (9). According to this tentative explanation, the reason why some patients develop one or another clinical presentation of the disease while displaying a broadly similar autoantibody profile could be related to the antibody titers and the tissue distribution of the primary autoantigen of aPLs, the plasma protein β2-glycoprotein-I (β2GPI) (10). According to murine models, the endothelial cells of the decidua express high amounts of β2GPI, making the decidua the primary target of aPLs. In contrast, endothelial cells of other vessels, monocytes, and platelets, which are responsible for the vascular manifestations of APS, function as secondary targets (11). Thus, even low titers of aPLs can bind to the decidua and trigger adverse pregnancy outcomes; however, only high titers of aPLs accompanied by an inflammatory stimulus, known as a “second hit,” can trigger vascular thrombosis (9, 12). According to the molecular mechanism that supports the two-hit hypothesis, a proinflammatory stimulus, such as LPS, would lead to the upregulation of TLR4 expression and the exposure of anionic phospholipids on the surface of endothelial cells. These molecules, in turn, perform as the primary receptors of the β2GPI/anti-β2GPI complexes (13).

While robustly supported *in vivo*, the two-hit hypothesis falls short of explaining the pathophysiology of APS thoroughly. On the one hand, it overlooks the existence of many aPLs that lack specificity for β2GPI (10, 14). On the other hand, adopting this hypothesis as the single explanation for APS would suggest that all patients with vascular APS also experience obstetric disturbances. Furthermore, *in vitro* findings indicate that aPLs can induce endothelial activation and dysfunction mechanisms without requiring a prior proinflammatory stimulus (15). Accordingly, we and other authors have described how IgG from APS patients induces, among other effects, decreased nitric oxide production in endothelial cells (15, 16), increased expression of adhesion molecules and tissue factor (TF) (17), and reduced expression of thrombomodulin (17). Nevertheless, it is not yet known whether these *in vitro* and second hit-independent mechanisms are sufficient to trigger the factual activation of the clot on the endothelial surface or the release of potentially procoagulant extracellular vesicles (EVs). This issue will be addressed in the present work.

## 2 Materials and methods

### 2.1 Sample collection and immunoglobulin G purification

Serum samples were collected from 11 APS patients who had both pregnancy-related morbidity and vascular thrombosis, and fulfilled the Sapporo/Sydney classification criteria (5) (APS group). Samples from 10 healthy women with proven gestational success (healthy control —HC— group) were also collected. Given that our research group previously identified differences in the mechanisms of aPL-induced endothelial damage and dysfunction related to treatment refractoriness (16), patients in the APS group were further classified for the coagulation assays according to their response to aspirin and heparin treatment as refractory (R) or non-refractory (NR). Recruitment of patients and healthy volunteers was conducted by the Recurrent Abortion Program of the Universidad de Antioquia, Colombia, and the Anticoagulation Clinic of Hospital San Vicente Fundación, Colombia, ensuring compliance with bioethical standards as approved by the respective bioethics’ committees (Official letters 006–2018 and 35-2018, respectively).

Pooled IgG samples were used to capture the representative effects of autoantibodies across different clinical APS groups, given that APS is characterized by a broad spectrum of aPLs with multiple potential recognizable epitopes, and that this heterogeneity is further compounded by considerable variability in autoantibody serum titers among individuals (18, 19). Pooled samples were subjected to protein G-based affinity chromatography (MAbTrap kit, GE Healthcare, Uppsala, Sweden) to purify immunoglobulin G (IgG). Purified IgG samples were characterized in terms of protein content by electrophoresis and spectrophotometry (NanoDrop, Thermo Fisher); the presence of aPLs, using commercial ELISA kits (Aeskulisa Phospholipid-8Pro-GM, Aesku diagnostics, Wendelsheim, Germany; Anti-cardiolipin antibodies ACA-IgG, Biosystems, Barcelona, Spain; IMTEC-β2-Glycoprotein I – Antibodies IgG, Magdeburg, Germany); and endotoxin detection, using an assay based on amebocyte lysate from Lymulus (Pierce LAL chromogenic endotoxin quantitation kit, Thermo Scientific, Rockford, IL, USA). These IgG samples have been previously used in our research group for *in vitro* assays. A complete characterization of all patients and healthy volunteers, as well as their respective IgG fractions, is presented in previous work (20, 21). A summary of their main clinical and serological features can be found in **Table 1**. IgG samples were consistently processed under sterile conditions, with endotoxin levels confirmed below 0.1 EU/mL (**Table 1**).

**Table 1:** Clinical characteristics of control and patient groups.

Clinical and laboratory characteristics	APS subgroups		APS (n = 11)	HC (n = 10)
	APS_R_ (n = 5)	APS_NR_ (n = 6)		
Age (mean ± standard deviation)	39.2 ± 8.41	38.83 ± 7.22	39.0 ± 7.38	35.5 ± 5.46
Pregnancy loss before the 10th wop (mean; maximum)	1.6; 5	1.2; 5	1.18; 5	0
Pregnancy loss after the 10th wop (mean; maximum)	2.7; 5	0.6; 2	1.45; 5	0
Deep venous thrombosis (n)	5	5	10	0
Pulmonary thrombo-embolism (n)	1	1	2	0
Systemic lupus erythematosus (n)	2	0	2	0
aβ2GPI (SGU) in serum GPL (mean ± SD) (positive > 20)	65.35 ± 45.7	67.79 ± 32.5	66.68 ± 36.93	2.29 ± 0.07
aβ2GPI (SGU) in purified IgG	65.2	85.3	75.3	0
aCL (GPL) in serum (mean ± SD) (positive > 10)	73.20 ± 41.2	93.73 ± 2.56	84.40 ± 28.26	0
aCL (GPL) in purified IgG	142	142.7	142.4	0
Endotoxins (EU/mL) in purified IgG. Detection limit 0.1	< 0.1	< 0.1	< 0.1	< 0.1

APS, patients with antiphospholipid syndrome; HC, healthy control (woman with proven gestational success); wop, weeks of gestation; aCL, anti-cardiolipin antibodies; aβ2GPI, anti-β2-glycoprotein-I antibodies; SGU, standard IgG units; GPL, standard units of anti-cardiolipin IgG; EU, endotoxin units.

### 2.2 Cell cultures

Umbilical cords from healthy donors were obtained under the consent of the mothers at local obstetric services (Hospital San Vicente Fundación, Medellín, Antioquia; Unidad Hospitalaria Metrosalud Manrique, Medellín, Antioquia). Primary human umbilical vein endothelial cells (HUVECs) were isolated from fresh cords using the protocol published by Jaffe et al. and standardized in our research group (22). Alternatively, HUVECs were commercially obtained (PromoCell, Heidelberg, Germany). HUVEC cultures were maintained in surface-treated flasks (Cellstar, Greiner Bio-One, Frickenhausen, Germany) until passage 4 (for fresh primary cells) or 12 (for commercial cells) at 37°C under a 5% CO_2_ atmosphere, with Endothelial Cell Growth Medium (ECGM, PromoCell). The medium was supplemented with the contents of the commercial supplement kit (PromoCell), 2% heat-inactivated fetal bovine serum (FBS) (or 10% FBS in the case of commercial HUVECs), and a 1% v/v antibiotic solution containing 5 mg/mL gentamicin (Genfar/Sanofi, Gentilly, France), 1,000 IU/mL penicillin G (Sigma-Aldrich, St. Louis MO, USA), and 25 μg/mL amphotericin B (Fungizone, Gibco, Grand Island NY, USA) for primary fresh HUVECs, or, for commercial HUVECs, 1% v/v of a commercial solution containing 10,000 IU/mL penicillin and 10,000 µg/mL streptomycin (PenStrep, Gibco, Grand Island NY, USA).

### 2.3 Assessment of IgG binding to HUVECs

HUVECs (8 x 10^4^ per well) were seeded in 24-well microplates (Sarstedt, Nümbrecht, Germany) and incubated at 37°C for 24 h. Once ~95% confluence was reached, the medium was replaced with ECGM supplemented with 20% FBS, and cells were pre-treated for 75 min at 37°C, with or without 0.5 µg/mL LPS. Thereafter, the medium was changed, and purified IgG (250 µg/mL) from APS patients or healthy controls was added for 2 h at room temperature, in the presence or absence of 0.5 µg/mL LPS. IgG was used at 250 µg/mL, a concentration previously shown in our group to reduce VEGF and nitric oxide production in HUVECs (16). LPS was applied at 0.5 µg/mL, high enough to support β2GPI antibody binding (13) but below the threshold we observed in preliminary experiments (≥4 µg/mL) at which LPS alone induced lEV release (data not shown).

After stimuli removal, cells were gently washed with 200 µL of blocking solution (2% w/v bovine serum albumin in phosphate-buffered saline —PBS—). Cells were stained by incubating for 30 min at room temperature with a 1:200 solution of anti-IgG antibody (Alexa Fluor 488 goat anti-human IgG, 2561903, Invitrogen, Eugene, USA), diluted in blocking solution. Finally, supernatants were removed, and cells were detached using 0.25% trypsin-EDTA. HUVECs were washed by centrifugation at 600 x g for 5 min at 4°C. Pellets were suspended in 200 µL of an ice-cold PBS containing 10% FBS and 0.1% sodium azide (NaN_3_). Immediately thereafter, 8 x 10^4^ events were analyzed on a flow cytometer (Accuri C6 Plus, BD, Franklin Lakes, USA) at a medium flow rate.

Alternatively, IgG binding to the endothelial cell surface was assessed using epifluorescence microscopy. For this approach, the same protocol was followed with some modifications: Before seeding the cells in the 24-well microplates, coverslips were inserted into the wells and were incubated overnight at 37°C with a 1:40 solution of collagen (Collagen Type I, Rat tail, Millipore, Massachusetts, USA) in PBS. HUVECs were fixed using 99% methanol for 5 min and stained with anti-human IgG antibody and DAPI (1:3000) for 2 h. Coverslips carrying the cells were removed from the bottom of the microplate wells and mounted on slides with a mounting medium (Vectashield Vibrance, Newark, USA). Slides were allowed to dry overnight at room temperature in the dark. Each sample was viewed under an epifluorescence microscope (Axio Imager A2, Oberkochen, Germany) with a constant exposure time. The entire diameter of each coverslip was manually scanned along the horizontal axis, and six fields were photographed at 40x magnification. The mean fluorescence of each image was analyzed.

### 2.4 Assessment of endothelial TF expression

HUVECs (8 x 10^4^ cells per well) were seeded in 24-well microplates and incubated at 37°C for 24 h, after which stimuli were added as described in the previous section. Wells were gently washed with PBS. HUVECs were detached using trypsin and washed in PBS by centrifugation at 240 × g for 3 min at 4°C. Cells were then suspended and incubated in a blocking buffer at room temperature for 10 min. A second wash was performed as described above. The pellets were stained in 100 µL of anti-Tissue Factor antibody solution (anti-CD142 Monoclonal Antibody APC, 17-1429-42, Thermo Scientific), at a 1:100 dilution in blocking buffer. Finally, HUVECs were suspended in 200 µL of ice-cold PBS containing 10% FBS and 0.1% NaN_3_. Immediately afterwards, 5 x 10^4^ events were acquired and analyzed using an Accuri C6 Plus flow cytometer at a medium flow rate.

### 2.5 Measurement of the endothelial surface coagulation activity

To estimate the surface coagulation activity of HUVECs, cell layers close to 95% confluence, composed of 3 x 10^4^ cells seeded in 96-well microplates and incubated for 24 h, were stimulated with purified IgG at 250 µg/mL (100 min) in 125 µL of ECGM supplemented with 2% FBS. As a negative control, the coagulation activity of untreated HUVECs was measured (non-treated cells; NTC). As a positive control, the cells were treated with 4 µg/mL LPS and 100 ng/mL phorbol myristate acetate (PMA).

To determine the role of the p38MAPK and MEK1/2 pathways in the coagulation activity of aPL-stimulated HUVECs, they were pre-treated with the specific inhibitors SB203580 at 20 µM (inhibitor of p38MAPK, Sigma-Aldrich, St. Louis MO, USA), and U0126 at 10 µM (inhibitor of MEK1/2, Abcam, Cambridge, UK), or an equivalent volume of dimethyl sulfoxide (DMSO) as vehicle for both inhibitors (Sigma-Aldrich, St. Louis MO, USA) for 1h before stimulation. Medium was removed and gently replaced with 50 µL fresh platelet-poor plasma (PPP) or fresh platelet-rich plasma (PRP). Plasma was obtained from blood samples collected in tubes containing sodium citrate from healthy individuals (men and women with no significant pathological or pharmacological history), whose prothrombin times were verified to be between 13.4 and 17.9 seconds. The microplate was incubated for 4.5 min in a water bath at 37°C with intermittent shaking.

Following this incubation, wells were recalcified by gently adding a pre-warmed 0.025 M CaCl_2_ solution. Then, a kinetic assessment of absorbance (λ = 405 nm) was performed every 7 min for approximately 12 h as an indirect measure of clot activation at the endothelial surface. The resulting coagulation curves were analyzed and compared according to the total absorbance difference before and after 12 h of recalcification. As additional controls, the plasma was recalcified in the presence of a 1:2 dilution of a commercial preparation of thromboplastin calcium (HemosIL, Bedford, MA, USA), or 0.25 µL enoxaparin at 10 IU/mL (Clenox, Pharmayect S.A., Bogotá, Colombia).

### 2.6 Enrichment of extracellular vesicles

EVs were enriched from the supernatants of aPL-stimulated HUVECs. Guided by previous assays, 1.5 x 10^6^ HUVECs were seeded in T75 culture flasks (Greiner Bio-One, Kremsmünster, Austria). The flasks were incubated at 37°C for 48 h until confluence reached approximately 95%. Stimulation with IgG was carried out in 5 mL ECGM supplemented with commercial EV-depleted FBS (ED-FBS, Gibco, Carlsbad, USA). The supernatants were recovered and subjected to centrifugation and ultracentrifugation. Briefly, to remove debris, samples were centrifuged at 380 x g for 10 min. The resulting supernatants were depleted of apoptotic bodies by a second centrifugation at 10,000 x g for 10 min. large EVs (lEVs) were then precipitated by centrifugation at 19,000 x g for 60 min. From the filtered supernatants of the last step (Pall Acrodisc PF 0.8/0.2 µm-pore-sized filters, Life Sciences), small EVs (sEVs) were enriched by ultracentrifugation at 100,000 x g for 120 min (Optima XPN-100 Ultracentrifuge, SW32Ti and SW41Ti rotors, Beckman Coulter, Brea, USA). Both lEVs and sEVs were washed through additional centrifugation in 10 mL sterile PBS, following the protocols previously employed for their precipitation. Finally, EVs were resuspended in approximately 200 µL PBS and stored at -80°C until their analysis.

### 2.7 Characterization of extracellular vesicles

EV samples were characterized using nanoparticle tracking analysis (NTA), cryo-transmission electron microscopy (cryo-TEM), and Western blotting (WB). For the NTA, the fractions containing the concentrated and washed EVs, along with their respective controls, were diluted in a 1:25 ratio in sterile PBS that had been previously filtered through 0.8/0.2 µm-pore-sized filters. The diluted samples were run through the instrument (Nanosight NS300, Malvern Panalytical, Worcestershire, UK) according to the manufacturer’s instructions. Control samples of EVs were measured, and the camera level, dilution factor, and threshold were set. In all cases, the particle count was verified to be between 10 and 100 particles per frame before calculating the absolute concentrations based on the volume in which the EVs were initially enriched. Also, the particle size distribution and mean diameter were assessed by NTA. For characterization by Cryo-TEM, 5 µL of each sample containing 2.63 x 10^6^ lEVs/µL or 1 x 10^7^ sEVs/µL were plunge-frozen in liquid ethane and transferred into a precooled CM120 Cryo-TEM (Philips, Eindhoven, Netherlands) using a Gatan 626-DH cryo-holder (Gatan, Pleasanton, USA). Micrographs were recorded with a 2 K CMOS Camera (F216 and EMMENU V4.0 software; camera and software TVIPS GmbH, Munich, Germany). Western blot analyses were performed by loading 5 μg of EV samples on a 4–12% precast SERVAGel™ gel (SERVA Electrophoresis GmbH, Heidelberg, Germany). Proteins were transferred to a polyvinylidene difluoride (PVDF) transfer membrane (Thermo Fischer Scientific GmbH, Rockford, USA), which was then blocked and immunoblotted. A complete description of the methods and reagents used for the analysis of EVs by Cryo-TEM and Western blotting can be found in our recent article (23).

IgG opsonization and CD54 cargo of lEVs were evaluated by flow cytometry as follows: 12 µL of enriched lEVs were incubated at room temperature for 15 min with 88 µL of 1X annexin V binding buffer (BD) diluted in commercial PBS and 1 µl of FITC-conjugated anti-CD54 (Immunotools, reference number 21279543S) or 1 µL of goat-anti-human IgG conjugated with AF488 (Invitrogen, catalog number A11013). After incubation, 200 μL of 1X annexin V binding buffer was added to each tube, and the samples were evaluated on an Accuri 6C Plus cytometer (BD) using reference beads (Megamix-Plus, BioCytex, reference number 7802).

### 2.8 Statistical analysis

Flow cytometry data were analyzed using FlowJo software (version 10.7.1, BD, Ashland, OR, USA). Further statistical analyses were performed in Prism 6.0.0 (GraphPad Software, San Diego, CA, USA). The assumption of normality was initially verified for each dataset using the Shapiro-Wilk hypothesis test. Groups were compared using two-way ANOVA or the repeated measures one-way ANOVA, followed by Tukey’s multiple comparison *post-hoc* test. For comparisons involving only two groups, paired t-tests were conducted. Differences with p < 0.05 were considered statistically significant. All data are presented as mean ± standard deviation.

## 3 Results

### 3.1 IgG from APS patients binds to HUVECs independently of an additional inflammatory stimulus

**Figure 1:**
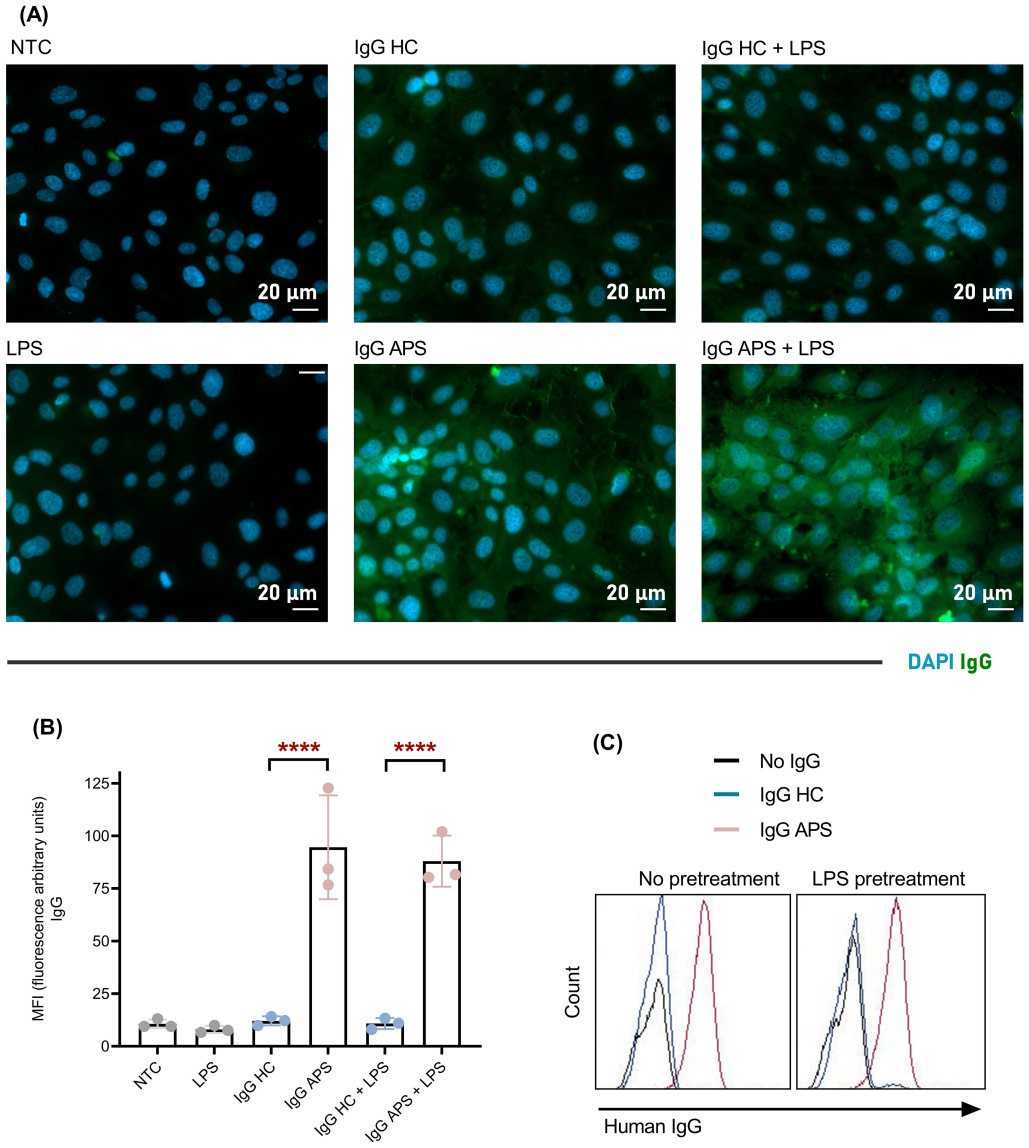
Epifluorescence microscopy and flow cytometry analysis demonstrating attachment of IgG APS to the surface of HUVECs independent of LPS. HUVECs were stimulated with IgG from APS patients or healthy controls in the presence or absence of a prior and concomitant stimulus with LPS. **(A)** Representative fluorescence images after anti-IgG antibody staining. **(B)** Mean fluorescence intensity, and **(C)** Representative histograms of HUVECs stained with fluorescent anti-IgG antibody, analyzed by flow cytometry (n = 3, repeated measures one-way ANOVA and Tukey’s multiple comparison test ****p < 0.0001). NTC, non-treated cells; LPS, lipopolysaccharide; IgG HC, IgG from healthy women with proven gestational success; IgG APS, IgG from women with APS; MFI, mean fluorescence intensity.

A key piece of evidence for the two-hit model is the *in vitro* finding that LPS stimulation of HUVECs induces TLR4 expression, thereby enabling the recognition of aβ2GPI/β2GPI complexes (13). To determine whether aPLs present in the IgG from APS patients can bind to HUVECs without the requirement of a prior proinflammatory stimulus, HUVECs were incubated with purified IgG both before and during stimulation with LPS at activating doses. In our experimental setting, IgG from APS patients binds to HUVECs even in the absence of LPS, as indicated by the green signals observed both in APS-only and APS + LPS treatments (**Figure 1A**). Moreover, this binding was significantly higher than that of IgG from healthy women with proven gestational success, both in the presence of LPS (HC, 10.77 ± 2.64; APS, 87.97 ± 12.15 MFI; p < 0.01) and its absence (HC, 12.08 ± 2.13; APS, 94.58 ± 24.67 MFI; p < 0.05) (**Figures 1B, C**).

### 3.2 Procoagulant activation of HUVECs by APS IgG involves p38MAPK and MEK1/2 pathways

**Figure 2:**
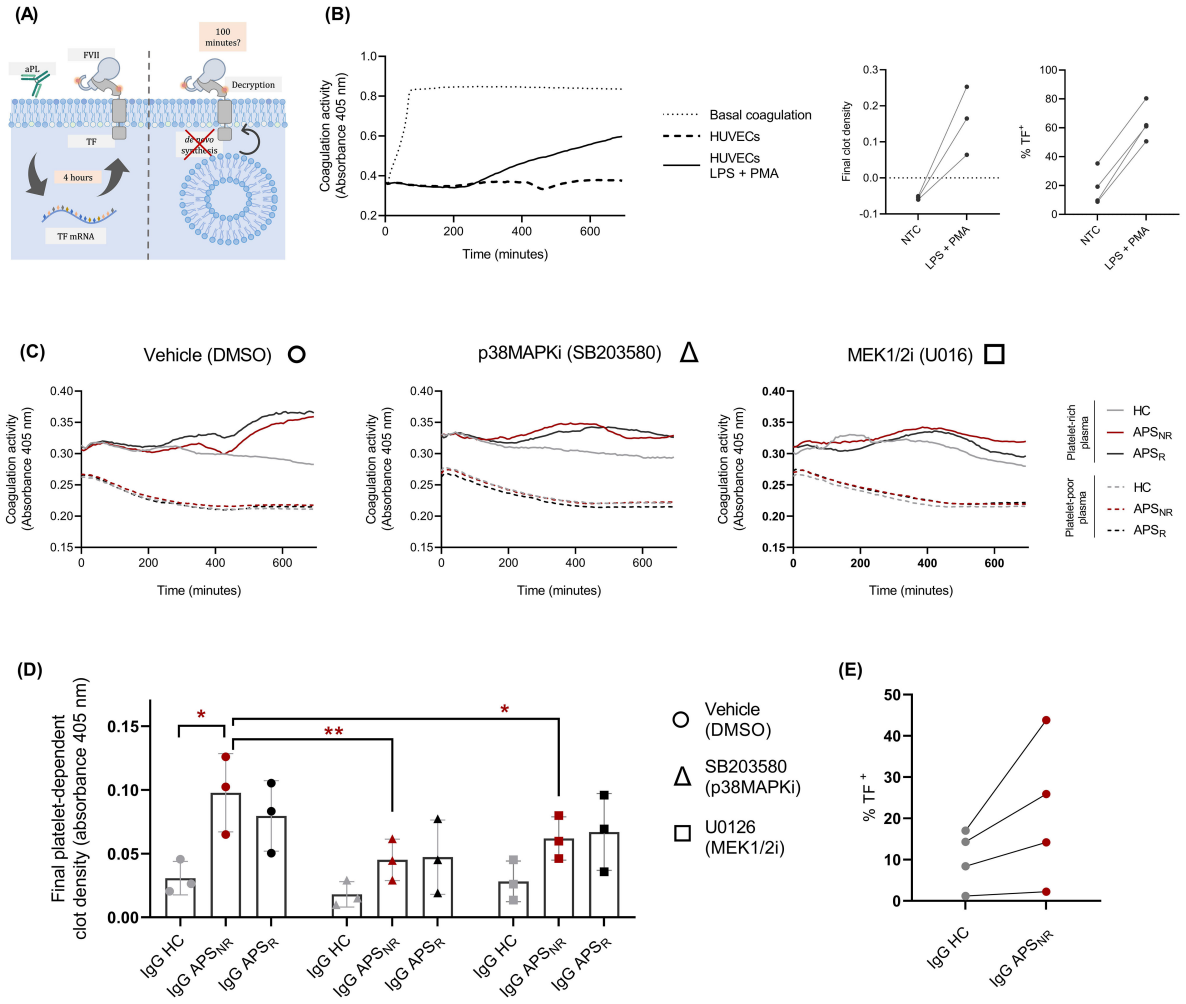
IgG from patients with APS enhances the procoagulant activity of HUVECs. **(A)** Temporal patterns of endothelial procoagulant activity: *de novo* TF synthesis (left) versus rapid TF mobilization (right) **(B)** Coagulation assay of recalcified PRP incubated without cells (Basal coagulation), with non-treated HUVECs, or with LPS+PMA-treated HUVECs. Left: Relative coagulation activity was assessed by measuring absorbance at 405 nm using a microplate spectrophotometer. Center: Clot density after 12 h of recalcification. Right: Flow cytometry evaluation of TF expression. **(C)** Coagulation assay of recalcified PRP (continuous line) or PPP (dashed lines) incubated with HUVECs pre-treated with DMSO (left), an inhibitor of the p38MAPK pathway (center), or an inhibitor of the MEK1/2 pathway (right). **(D)** Platelet-rich plasma-dependent clot density after 12 h of recalcification in the presence of the inhibitors SB203580, U0126, or their vehicle (DMSO) (ANOVA and Tukey’s multiple comparison test). **(E)** Flow cytometry evaluation of TF expression in HUVECs (paired t-test). (In all cases, n = 3 - 4, *p < 0.05, **p < 0,01, ***p < 0.001, ns not significant). NTC, non-treated HUVECs; LPS + PMA, lipopolysaccharide plus phorbol ester; PRP, platelet-rich plasma; PPP, platelet-poor plasma; NR, no-refractoriness to treatment; R, refractoriness to treatment; SB203580, p38MAPK inhibitor; U0126, MEK1/2 inhibitor; TF, tissue factor.

Since TF gene and surface expression induced by aPLs require at least 2 h of stimulation and continues to increase up to 6 h (17, 24), a 100-min activation period was selected to avoid the onset of transcription-dependent effects. Within this timeframe, increases in TF activity predominantly reflect the rapid, protein synthesis–independent process of TF decryption previously described in the presence of platelets (25) (**Figure 2A**). Using this short stimulation window, HUVECs treated with LPS or PMA displayed a pronounced increase in procoagulant activity in PRP after 100 min of treatment, followed by 12 h (760 min) of plasma recalcification (**Figure 2B**). A similar response was observed in PPP (data not shown), indicating that clot formation under these conditions does not depend on platelets.

Under the same stimulation conditions, HUVECs exhibited increased clotting activity when treated with IgG from non-refractory APS patients (APS_NR_), compared to stimulation with IgG from the HC group (HC, 0.031 ± 0.013 ΔA; APS_NR_, 0.098 ± 0.031 ΔA; p < 0.01), specifically in PRP. This increase in coagulation activity was partially attenuated by chemical inhibition of the p38MAPK and MEK1/2 pathways (APS_NR_, 0.098 ± 0.031 ΔA; APS_NR_ + SB203580, 0.045 ± 0.016 ΔA; APS_NR_ + U0126, 0.062 ± 0.017 ΔA; p < 0.05) (**Figures 2C, D**), depended on the presence of PRP and was accompanied by a higher proportion of TF-positive cells in four independent replicates (HC, 10.23 ± 7; APS_NR_ 21.53 ± 17.72%; ns) (**Figure 2E**). A similar trend was observed with IgG from APS_R_; however, the differences did not reach statistical significance. No differences in TF expression were detected between HUVECs treated with IgG from the HC group and those treated with IgG from the APS_R_ group (data not shown).

### 3.3 APS IgG induces the release of large extracellular vesicles

**Figure 3:**
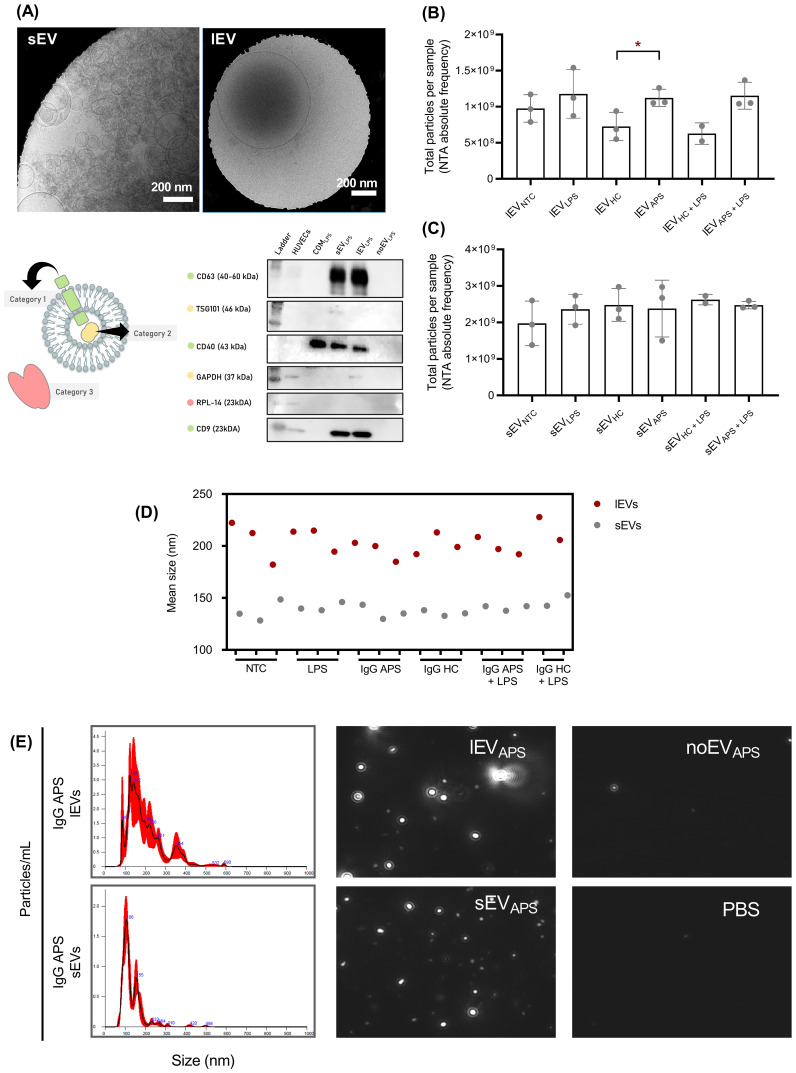
IgG from APS patients induces an increase in the release of lEVs in HUVECs. **(A)** Cryo-TEM micrographs (top) and Western blot analysis (bottom) of EV fractions enriched from HUVEC supernatants. Three different categories of markers are graphically symbolized. Category 1, membrane proteins; Category 2, soluble proteins that are loaded within EVs; Category 3, negative controls (proteins not associated with EVs). **(B)** Adjusted quantification of total lEVs and **(C)** sEVs, as determined by nanoparticle tracking analysis (n = 3, t-test, *p < 0.05). **(D)** Comparison of the mean size of lEVs and sEVs. **(E)** Representative images of nanoparticle tracking analysis. lEV, large extracellular vesicles; sEV, small extracellular vesicles; NTC, non-treated cells; LPS, lipopolysaccharide; HC, IgG from healthy women with proven gestational success; APS, IgG from patients with APS; noEV, extracellular vesicle-depleted supernatant fraction; COM, complete enrichment matrix (HUVECs supernatant without enrichment in EVs).

Release of EVs from endothelial cells may contribute to the hypercoagulable state of plasma in APS. To identify the optimal EV harvesting time and minimize recapture, HUVECs were stimulated with IgG at multiple time points between 2 and 48 h, revealing two activation peaks at 4 and 12 h (**Supplementary Figure S1**). Based on this, EVs were collected after 12 h of stimulation in three consecutive rounds, totaling 36 h. The identification of spherical structures smaller than 100 nm (sEVs) and between 100 and 500 nm (lEVs) through Cryo-TEM, as well as the presence of key markers evidenced in the WB, indicate the enrichment of two EV subpopulations (**Figure 3A**). HUVECs treated with IgG from APS patients released higher amounts of lEVs compared to those treated with IgG from healthy controls (HC, 7.3 x 10^8^ ± 2 x 10^8^; APS, 1.1 x 10^9^ ± 1.2 x 10^8^ lEVs; p < 0.05) (**Figure 3B**). No significant differences were observed in the release of sEVs (**Figure 3C**). Additional stimulation with LPS did not alter these results. Controls of the EV enrichment process, including size distribution according to NTA, are also shown (**Figures 3D, E**).

### 3.4 EVs released from HUVECs upon APS IgG are opsonized and carry adhesion molecules

**Figure 4:**
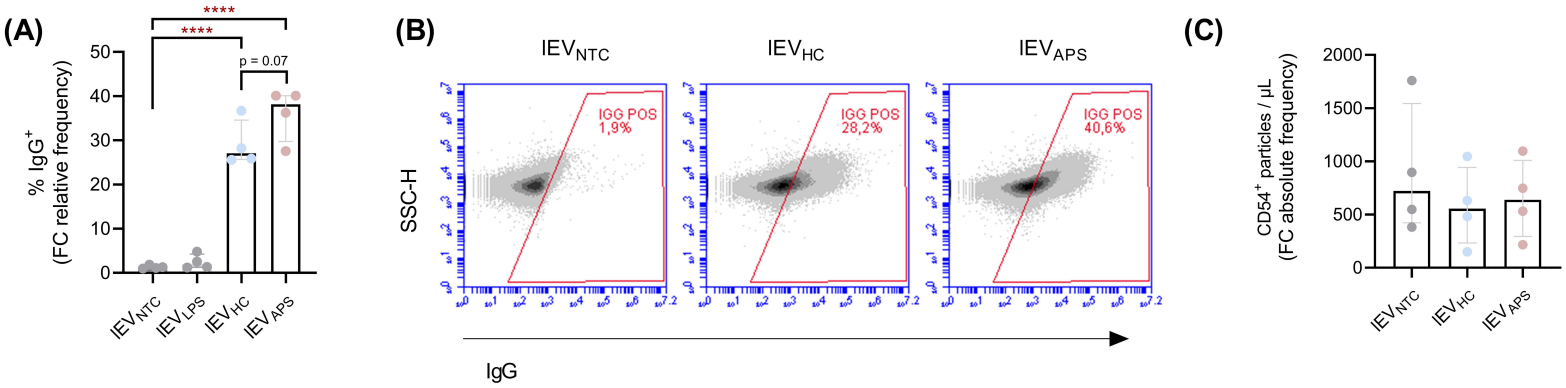
IgG from APS patients induces the release of lEVs that are opsonized by IgG. HUVECs were stimulated with IgG from APS patients or control women. Conditioned media were depleted of detritus, and lEVs were enriched by ultracentrifugation. lEVs were assessed by flow cytometry. **(A)** Percentage of lEVs exhibiting human IgG on their surface and **(D)** representative dot plots (n = 4, unpaired t-test, ****p < 0.0001). **(C)** Concentration of particles exhibiting CD54 on their surface in the supernatant enriched in lEVs (n = 4, one-way ANOVA, Tukey’s multiple comparison test). lEVs, large extracellular vesicles; IgG, immunoglobulin G; HC, control volunteers; APS, antiphospholipid syndrome patients; NTC, non-treated cells; LPS, lipopolysaccharide.

To assess the potential contribution of lEVs to immune and vascular cell interactions in APS, CD54 and IgG were measured on the lEV surface by flow cytometry. A greater proportion of lEVs released from HUVECs upon APS IgG stimulation (lEV_APS_) were opsonized with human IgG compared to those released after stimulation with IgG from healthy controls (lEV_HC_) (lEV_HC_ 29.11%; lEV_APS_ 36.01%; p = 0.07), whereas EVs from untreated cells rarely carried IgG (~2%) (**Figures 4A, B**). In contrast, the proportion of lEVs positive for the endothelial adhesion molecule CD54 was low in all conditions (between 1-2%) and not significantly different from that observed in EVs from untreated HUVECs. While the absolute number of CD54^+^ lEVs per µL was slightly higher in HUVECs stimulated with APS IgG compared with control IgG, this difference was not statistically significant (lEV_HC_, 576.9; lEV_APS_, 647.4; p= 0.416) (**Figure 4C**).

## 4 Discussion

Although *in vivo* results suggest that the thrombotic effects of aPLs require a prior stimulus (9, 12), and *in vitro* assays using monoclonal aβ2GPI antibodies reinforce this hypothesis (13), our findings indicate that the aPLs present in IgG from APS patients can bind to the endothelial surface, enhance its coagulation activity, and lead to the release of lEVs that carry adhesion molecules and become opsonized without requiring an additional “second hit”.

The results from the intravascular administration of IgG from APS patients to rats are robust and strongly support the role of LPS as an additional condition for developing thrombosis (9, 11, 12). Consequently, the results reported here should not to be taken as evidence that the second hit can be omitted in aPL-mediated vascular thrombosis. Instead, our findings indicate that refinement of the two-hit hypothesis is warranted by incorporating additional factors and exploring new molecular mechanisms, especially in the context of obstetric APS.

Obstetric APS manifestations occur even in the presence of low autoantibody titers (10), possibly because β2GPI is abundant in the decidual endothelium (11). In contrast, vascular manifestations require high autoantibody titers together with a preceding inflammatory stimulus, which leads to the exposure of TLR4 on the surface of endothelial cells and, thus, enables the binding of aβ2GPI/β2GPI complexes (13). Clinical evidence from this and other previous studies, including those from our group and others, supports this concept, as patients with isolated obstetric APS typically exhibit low titers of criteria aPLs. This observation has even contributed to the formulation of new diagnostic categories, such as seronegative obstetric APS (19, 21, 26, 27).

However, this explanation 1) assumes that all patients with vascular manifestations also exhibit obstetric complications and 2) overlooks the diversity of aPLs in terms of antigen specificity and avidity, focusing exclusively on antibodies against β2GPI. These aspects could refine the two-hit hypothesis. Patients with vascular APS may correspond to those with high-avidity antibodies or with antigenic specificities capable of binding to the resting endothelial surface through alternative mechanisms. This pattern could account for the broad clinical spectrum observed in aPL-positive individuals: while some exhibit medium to high aPL titers without clinical manifestations, others develop severe thrombosis, catastrophic APS episodes, or recurrent miscarriages despite treatment with aspirin and low-molecular-weight heparin (28).

Lupus anticoagulant (LA) antibodies may be related to this increased pathogenicity profile. As previously reported, LA independently predicts clinical manifestations (29). The unique feature of the LA test is that it measures a functional capacity, rather than detecting antibodies with a specific antigenic specificity, which may be relevant to the pathophysiology of the disease. Paradoxically, this functional capacity consists of prolonging *in vitro* clotting times (30). Notably, in recent years, LA activity has been attributed to antibodies with antigen specificity for phosphatidylserine/prothrombin complexes (aPS/PT) (31, 32). Moreover, evidence from a patient cohort suggests that the detection of aPS/PT may help to predict both vascular thrombosis and gestational morbidity (33). However, the effects of these antibodies within the framework of the two-hit hypothesis have not yet been studied.

Mechanistically, anti-β2GPI antibodies are the most thoroughly characterized in terms of the mechanisms by which they induce endothelial activation and vascular thrombosis. Anti-β2GPI antibodies are known to inactivate endothelial nitric oxide synthase (eNOS) via binding to the apoprotein E receptor 2 (ApoER2) on endothelial cells (15, 17). Likewise, these and other aPLs can lead to the activation of protein kinase B and the mammalian target of rapamycin (mTOR) pathway, which has been associated with hyperplasia of the tunica intima (34, 35). In addition to binding to TLR4, aPLs can also be internalized by endosomes and, through activation of endosomal NADPH oxidase (NOX), they activate the nuclear factor kappa-light-chain-enhancer of activated B cells (NF-κB) pathway, leading to the expression of TF and adhesion molecules such as CD62E, CD106, and CD54 (17, 36, 37). The increase reported here in the procoagulant activity on the endothelial cell surface mediated by IgG APS, particularly observed in the APS_NR_ group, could be linked to these previously reported APS-related mechanisms, including TF overexpression, reduced thrombomodulin expression, and decreased nitric oxide (NO) release (15, 17, 37). In line with this, our previous work showed that IgG from both refractory and non-refractory APS patients reduces NO production in HUVECs, although the activation effect was more pronounced with APS_NR_ IgG (lower production of VEGF and greater release of lEVs compared to IgG HC) (16), reflecting differences in endothelial activation and coagulant activity between the two patient subgroups. This reduction in NO may also favor platelet activation (38) and, consequently, enhance the observed platelet-dependent procoagulant activity in APS_NR_ IgG-treated HUVECs. Furthermore, the procoagulant activity observed in APS_NR_ IgG-treated endothelial cells was partially attenuated by inhibition of the p38MAPK and MEK1/2 pathways, highlighting the critical role of these signaling cascades in mediating endothelial activation and thrombosis, even in the absence of a proinflammatory stimulus. These findings support the notion that the molecular mechanisms driving vascular thrombosis in APS are complex and involve multiple signaling pathways, with significant contributions from platelet activation and endothelial dysfunction.

Another relevant aspect is the release of lEVs from aPL-stimulated endothelial cells. Patients with aPLs exhibit increased plasma lEVs, consistently of endothelial origin (reviewed in (39)). Although evidence that aPLs directly induce lEV release in endothelial cells is limited (40), our results support this notion. Furthermore, our findings demonstrate that lEVs derived from IgG-treated HUVECs carry CD54 and IgG on their surface, with IgG levels being enhanced following treatment with APS IgG. The presence and possible transfer of these molecules to other cell types may have important functional implications: CD54 can induce the expression of TF in monocytes (41), while surface-bound IgG could enable the formation of immune complexes capable of delivering procoagulant or inflammatory signals to phagocytes (42). Consistent with these observations, we reported previously that lEV_APS_ have procoagulant activity, which can be attenuated by LA-like antibodies (20). Further, lEV_APS_ of endothelial origin are also known to carry the adhesion molecule CD62E (40), and plasma EVs from patients with LA have a lower presence of phosphatidylserine and β2GPI on their surface (42). The lack of significant changes induced by sEVs in our study may reflect differences in EV biogenesis, a lower content of procoagulant or inflammatory molecules, or a more modulatory role of sEVs in intercellular communication. It is also possible that the small sample size used in this study limited the detection of some effects, highlighting the need for further investigation.

The choice of HUVECs over microvascular or arterial endothelial cells is based on two considerations: the predominance of thrombotic events in APS patients affecting the venous segment (43), and previous studies using HUVECs that illustrate different scenarios regarding the role of a potential “second hit” in endothelial activation. While monoclonal aβ2GPI antibodies (IS3 and 2F3) were reported to bind efficiently to HUVECs only in the presence of LPS-induced activation (13), polyclonal IgG from APS patients was shown to induce lEV release without additional inflammatory signals (40). Our results align with the latter, suggesting that endothelial activation by aPLs translates into increased surface procoagulant activity, with lEVs released in this context carrying CD54 and opsonized by human IgG. Similar mechanisms have been observed in other endothelial cells including cerebral microvasculature and aortic endothelial cells, where APS serum binds to the endothelium (44). In these models, IgG treatment increases endogenous thrombin, TF expression, and decreases thrombomodulin (17), suggesting these effects may extend beyond venous endothelium, although further studies are required to confirm this.

While relevant insights are offered by the present findings, they must be interpreted within the conceptual and technical boundaries of the model used. IgG pools were chosen due to the significant heterogeneity in antigenic specificity and antibody titers among patients. This strategy was intended to minimize individual variability and highlight immunological effects that may be generalizable across patients with shared clinical characteristics. It should be acknowledged that this approach requires complementation by studies using individual IgG samples or well-characterized monoclonal antibodies to confirm whether specific autoantibodies can activate endothelial cells independently of a second hit.

In conclusion our results suggest additional factors that may refine the classical second-hit hypothesis derived from murine models of vascular APS. Certain aPL profiles in APS may have intrinsic pathogenic potential, particularly independent of a second hit. The partial inhibition of coagulation by p38MAPK and MEK1/2 blockers highlights key signaling pathways that modulate this process. Further, the release of EVs that subsequently display surface IgG may also be biological relevant. Overall, these findings expand the classical second-hit hypothesis by demonstrating that endothelial-intrinsic mechanisms and EV release may contribute to thrombotic risk in APS.

## Data Availability

The original contributions presented in the study are included in the article/**Supplementary Material**. Further inquiries can be directed to the corresponding authors.
