# Correction: Antiphospholipid antibodies induce endothelial procoagulant activity and release of extracellular vesicles independently of a second hit

**DOI:** 10.3389/fimmu.2026.1816625

**Published:** 2026-04-22

**Authors:** Daniel Álvarez, Hephzibah E. Winter, Udo R. Markert, Ángela P. Cadavid J., Diana M. Morales-Prieto

**Affiliations:** 1 Grupo Reproducción, Departamento Microbiología y Parasitología, Facultad de Medicina, Universidad de Antioquia UdeA, Medellín, Colombia; 2 Placenta Lab, Department of Obstetrics, Jena University Hospital, Jena, Germany; 3 Facultad de Enfermería, Universidad Cooperativa de Colombia (UCC), Medellín, Colombia; 4 Grupo de Investigación en Trombosis, Departamento Medicina Interna, Facultad de Medicina, Universidad de Antioquia UdeA, Medellín, Colombia

**Keywords:** antiphospholipid antibodies, antiphospholipid syndrome, hemostasis, endothelial cells, extracellular vesicles, β2-glycoprotein-I

The amount of protein used in the Western Blot was unintentionally misreported. This error does not alter the meaning of our results in any way.

A correction has been made to the section 2 **Materials and methods**, 2.7 *“Characterization of extracellular vesicles*”, paragraph 1:

“Western blot analyses were performed by loading 5 µg of EV samples on a 4–12% precast SERVAGel™ gel (SERVA Electrophoresis GmbH, Heidelberg, Germany)”.

In the **Acknowledgements**, two of our collaborators were inadvertently omitted.

The correct **Acknowledgements** is as follows:

“We sincerely thank all the women who selflessly contributed to this study with their blood samples and clinical data. We would like to thank Paulina Fuentes Zacarias, PhD student at the Placenta Lab Jena, for performing the Western blot analyses of the EVs, and Frank Steiniger and his team at the Elektronenmikroskopisches Zentrum Jena for performing the CryoTEM of the EVs”.

The original version of this article has been updated.

