# Supplementary figures and images for: Antiphospholipid antibodies induce endothelial procoagulant activity and release of extracellular vesicles independently of a second hit
Source: Front Immunol. 2025 Dec 16;16:1702103. doi: 10.3389/fimmu.2025.1702103 (PMC12750021; doi:10.3389/fimmu.2025.1702103)

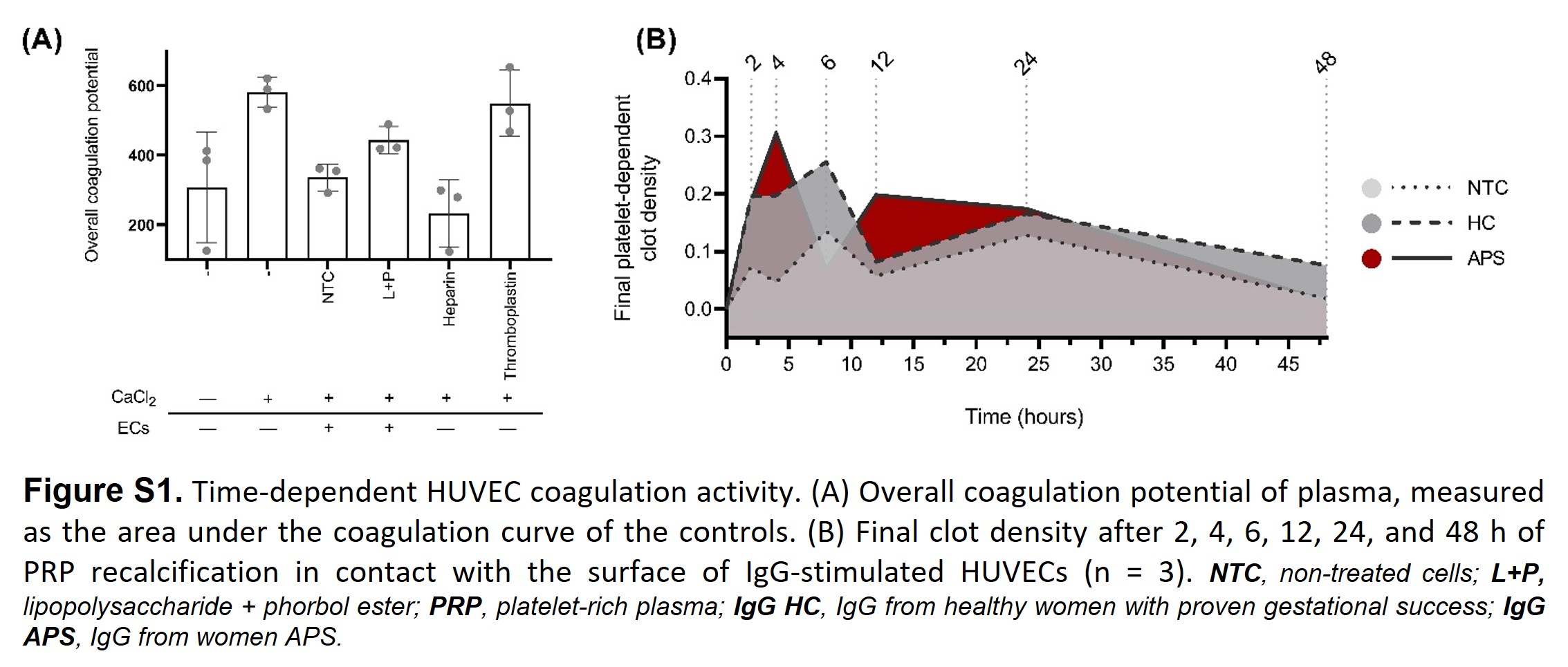

Supplement: Supplementary file 1 [file Image1.jpeg]
